# Diabetes mellitus and cardiac complications in thalassemia major patients

**DOI:** 10.1186/1532-429X-14-S1-P189

**Published:** 2012-02-01

**Authors:** Alessia Pepe, Antonella Meloni, Vincenzo Caruso, Paolo Cianciulli, Elisabetta Chiodi, Gennaro Restaino, Vincenzo Positano, Petra Keilberg, Massimo Lombardi, Maria Rita Gamberini

**Affiliations:** 1 CMR Unit, Fondazione G.Monasterio CNR-Regione Toscana and Institute of Clinical Physiology, Pisa, Italy; 2 Unità Operativa Dipartimentale Talassemia, P.O. "S. Luigi-Currò" - ARNAS Garibaldi, Catania, Italy; 3 Centro Talassemie, Ospedale "Sant'Eugenio Papa", Roma, Italy; 4 Dipartimento di Radiologia, Ospedale “Sant’Anna”, Ferrara, Italy; 5 Pediatria, Adolescentologia e Talassemia, Ospedale “Sant’Anna”, Ferrara, Italy; 6 Departement of radiology, ‘‘John Paul II’’ Catholic University, Campobasso, Italy

## Background

The relationship between diabetes mellitus (DM) and cardiac complications has never been systematically studied in thalassemia major (TM). The aim of this cross-sectional study was to evaluate in a large historical cohort of TM in the cardiovascular magnetic resonance (CMR) era if DM was associated with an higher prevalence and risk of heart complications, also regardless to the presence of myocardial iron overload (MIO).

## Methods

We compared 86 TM patients affected by DM with 709 TM patients without DM enrolled in the Myocardial Iron Overload in Thalassemia (MIOT) network at their first CMR. The MIOT network involves 68 thalassemia centers and 8 CMR sites where the exams are performed using standardized and validated procedures and all centers are linked to a web-based database to collect and share patients' history, clinical and diagnostic data.

Cardiac iron was evaluated by T2* multiecho multislice technique. Biventricular function parameters were quantitatively evaluated by cine images. Myocardial fibrosis was evaluated by late gadolinium enhacement CMR technique.

Heart failure (HF) was diagnosed by CMR in presence of a left ventricular (LV) and/or right ventricular (RV) ejection fraction (EF) lower than 4 standard deviations from the mean value normalized by sex and age. Moreover HF was identified by patients’ history, diagnosed by clinicians based on symptoms, signs and echocardiographic findings. All considered cardiac events were developed after the DM diagnosis.

## Results

In DM patients versus no-DM patients we found a significantly higher prevalence of cardiac complications (HF, arrhythmias and pulmonary hypertension) (46.5% vs 16.9%, P<0.0001), HF (30.2% vs 11.7%, P<0.0001), hyperkinetic arrhythmias ECG documented and requiring medications (18.6% vs 5.5%, P<0.0001), and myocardial fibrosis (29.9% vs 18.4%, P=0.008).

To evaluate the impact of the DM on cardiac complications also in relationship to MIO, we always adjusted the risk of cardiac findings for the absence of MIO (all segments with T2*≥20 ms). Patients with DM had a significant higher risk of cardiac complications (OR 2.84, P<0.0001), HF (OR 2.32, P=0.003), hyperkinetic arrhythmias (OR 2.21, P=0.023) and myocardial fibrosis (OR 1.91, P=0.021).

## Conclusions

DM increases the risk for cardiac complications, HF, hyperkinetic arrhythmias and myocardial fibrosis irrespective of MIO.

## Funding

“No-profit” support by industrial sponsorships (Chiesi, Apotex and GE Healtcare) and “Ministero della Salute, fondi ex art. 12 D.Lgs. 502/92 e s.m.i., ricerca sanitaria finalizzata anno 2006” e “Fondazione L. Giambrone”.

**Table 1:** Logistic regression analysis: ORs (95% CI) of DM versus no-DM patients for cardiac findings adjusted for the covariates significantly different between groups and significantly associated to the dependent variable.

	Adjusted for no-MIO		Adjusted for no-MIO and covariates	
	**OR (95% CI)**	**P-value**	**OR (95% CI)**	**P-value**

***Cardiac complications***	4.23 (2.65-6.76)	<0.0001	2.84 (1.71-4.69) #	<0.0001
***Heart failure***	3.14 (1.87-5.26)	<0.0001	2.33 (1.33-4.06) #	0.003
***Heart failure at time of study***	3.45 (2.02-5.89)	<0.0001	2.48 (1.39-4.43) #	0.002
***Hyperkinetic arrhythmias***	4.09 (2.16-7.74)	<0.0001	2.21 (1.12-4.37) #	0.023
***Myocardial fibrosis***	2.12 (1.24-3.63)	0.006	1.91 (1.11-3.29) §	0.021
***Heart dysfunction (LV and/or RV)***	1.45 (0.37-2.33)	0.093		
***Biventricular dysfunction***	1.46 (0.78-2.72)	0.235		
***LV dysfunction***	0.77 (0.37-1.60)	0.487	1.62 (0.44-5.97) *	0.470
***RV dysfunction***	1.82 (1.01-3.30)	0.048	1.33 (0.71-2.49) *	0.366

Covariates: * age; § endocrine co-morbidity; # age and endocrine co-morbidity.

**Figure 1:**
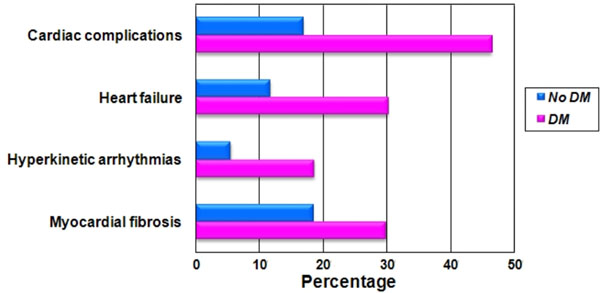
Prevalence of significant cardiac end-points for patients with and without DM.

